# Analysis of risk factors for infant mortality in the 1992-3 and 2002-3 birth cohorts in rural Guinea-Bissau

**DOI:** 10.1371/journal.pone.0177984

**Published:** 2017-05-18

**Authors:** Stine Byberg, Marie D. Østergaard, Amabelia Rodrigues, Cesario Martins, Christine S. Benn, Peter Aaby, Ane B. Fisker

**Affiliations:** 1 Bandim Health Project, Indepth Network, Bissau, Guinea-Bissau; 2 Research Center for Vitamins and Vaccines (CVIVA), Bandim Health Project, Statens Serum Institut, Copenhagen, Denmark; 3 OPEN, Odense Patient data Explorative Network, Odense University Hospital/Institute of Clinical Research, University of Southern Denmark, Odense, Denmark; Universidade Nova de Lisboa Instituto de Higiene e Medicina Tropical, PORTUGAL

## Abstract

**Introduction:**

Though still high, the infant mortality rate in Guinea-Bissau has declined. We aimed to identify risk factors including vaccination coverage, for infant mortality in the rural population of Guinea-Bissau and assess whether these risk factors changed from 1992–3 to 2002–3.

**Methods:**

The Bandim Health Project (BHP) continuously surveys children in rural Guinea-Bissau. We investigated the association between maternal and infant factors (especially DTP and measles coverage) and infant mortality. Hazard ratios (HR) were calculated using Cox regression. We tested for interactions with sex, age groups (defined by current vaccination schedule) and cohort to assess whether the risk factors were the same for boys and girls, in different age groups in 1992–3 and in 2002–3.

**Results:**

The infant mortality rate declined from 148/1000 person years (PYRS) in 1992–3 to 124/1000 PYRS in 2002–3 (HR = 0.88;95%CI:0.77–0.99); this decline was significant for girls (0.77;0.64–0.94) but not for boys (0.97;0.82–1.15) (p = 0.10 for interaction). Risk factors did not differ significantly by cohort in either distribution or effect. Mortality decline was most marked among girls aged 9–11 months (0.56;0.37–0.83). There was no significant mortality decline for girls 1.5–8 months of age (0.93;0.68–1.28) (p = 0.05 for interaction). DTP and measles coverage increased from 1992–3 to 2002–3.

**Conclusions:**

Risk factors did not change with the decline in mortality. Due to beneficial non-specific effects for girls, the increased coverage of measles vaccination may have contributed to the disproportional decline in mortality by sex and age group.

## Introduction

The fourth Millennium Development Goal (MDG4) was to reduce under-five mortality by two-thirds between 1990 and 2015. Infant mortality constitutes approximately 75% of the under-five mortality [1]. Although child mortality has declined globally, the decline has been strongest among older children and in the most developed regions of the world [1]. Under-five mortality failed to decline as rapidly in Sub-Saharan Africa as in other regions, and Sub-Saharan Africa still accounted for approximately half of all child deaths worldwide in 2011 [2]. It is therefore important that factors related to mortality decline be identified in order to prioritize efforts aimed at reducing child mortality even further and to ensure that the factors are not removed which could cause subsequent increases in mortality.

Guinea-Bissau, a small country in West Africa, has the seventh highest infant mortality rate in the world [3]. However, child mortality has declined during the last decades. The Bandim Health Project (BHP) in Guinea-Bissau continuously surveys a random sample of children living in the rural areas, and has done so since 1990. Thus, it could be documented that in these areas the infant mortality rate dropped from 192 per 1000 in the early 1990’s [4], to 135 per 1000 in the 2003–5 [5]. It has been suggested that the observed decline in mortality may be due to a drop in the malaria prevalence [6, 7], or improved coverage of routine vaccinations or even the increasing number of campaigns with vaccines and vitamin A supplementation [8, 9]. However, the main reasons for the decline in mortality have not been determined.

The aim of the present study was to identify the most important risk factors for infant mortality and to examine whether changes in these risk factors could explain the decline in mortality. Identification of factors related to mortality decline could aid in further reduction of infant mortality. During the high mortality era, several risk factors for infant mortality have been identified, including low maternal education, low maternal age, and short birth spacing [10–12]. It is unknown whether these risk factors are still important with declining mortality. We specifically examined whether the mortality rates and risk factors differed by age group and sex as coverage of childhood interventions are strongly associated with age, and sex modifies the effect of many childhood interventions [13]. In addition, we examined vaccination coverage to assess whether changes in coverage could explain the decline in mortality.

## Methods

### Setting

BHP has been working in Guinea-Bissau for more than 35 years, conducting research based on a routine demographic surveillance system. The demographic surveillance system was established in five regions in rural Guinea-Bissau in 1990. One hundred clusters, each including 100 women of reproductive age and their children under five years of age, were randomly selected and followed-up every 6 months by mobile teams from BHP. New pregnancies, new children under five who were either born or moved into the study area are registered at all visits. Information on place of delivery (home, health facility), antenatal care and who assisted the delivery, is collected at first visit after the birth of a child. Women living in the study areas are registered at 14–16 years of age and followed until they move out of the area or die. At registration, women are interviewed about past obstetric history, age, ethnicity, tetanus vaccinations and whether they have attended school. For all children below 5 years of age, information for assessing child health status is collected at each visit, including vital status, vaccinations, use of bed nets and hospitalisations.

### Risk factors

We compared the distribution of the variables measured for both cohorts and prospectively assessed the association with mortality for the birth cohorts 1992–1993 and 2002–2003. We chose the first period as early as possible after the initiation of the data collection, allowing for a “honey-moon period” to assure that the data collection was running well in all regions and had been doing so for a while. The second period was chosen to avoid the period from 2005 and onward, when randomised trials of community based malaria treatment and vitamin A supplementation were underway [14].

We studied the following risk factors which were assessed at the beginning of the surveillance period; sex of the child, region, maternal ethnicity and education, season of birth (rainy season (June-November) vs. dry season (December-May)), maternal age at birth of the child, previous loss of child, if the child was delivered with skilled attendance, born at a health care facility, distance to nearest health facility, number of siblings and twin status. Information on antenatal care attendance was not collected in 1992–3 and is therefore not included in the present study. Likewise, we did not include maternal tetanus vaccination as we did not have this information at birth for all children and the information may therefore be subject to bias.

During the study period, BCG vaccination and oral polio vaccine (OPV) were recommended at birth, diphtheria-tetanus-pertussis vaccine (DTP) and OPV at 6, 10 and 14 weeks of age, and measles vaccine (MV) at 9 months [15]. We assessed vaccination coverage in the two birth cohorts, by assessing coverage at the particular age using information collected during the subsequent 12 months. For example, coverage by 2 months of age was assessed among all children aged 2–13 months at time of health card inspection. Only children with a health card seen during the one year span were included in the coverage estimates. Furthermore, we calculated the proportion of children who had DTP as last vaccine, DTP and MV co-administered as last vaccine or MV-only as last vaccine.

### Statistical analyses

Mortality rates were compared in Cox regression models investigating the influence of maternal and infant risk factors on subsequent death in both univariate and multivariate analyses in 1992–1993 and in 2002–2003, respectively. All live born children contributed time at risk from the date of birth (or date of registration if identified after birth) until death, migration or 1 year of age whichever came first. We also conducted sub-analyses of risk factors by age groups; 0–45 days of life, 1.5–8 months, and 9–11 months, corresponding to the three major vaccination ages for BCG, DTP and MV, respectively. When studying the mortality by age groups, the child contributed time at risk from the beginning of the interval/date of registration (whichever came last) until the end of the interval considered. Interactions between risk factors and sex were also investigated.

In the combined dataset of the two birth cohorts, we investigated whether the risk factors had changed over time in the two cohorts. We did this by parameterising the model with risk factor(s), cohort and a term for interaction between the risk factor(s) and cohort both in the univariate and in the multivariate analyses. Furthermore, we assessed if the association between the risk factor(s) and mortality had changed over time.

The variables maternal age and distance to health facility/distance to nearest hospital were tested for a linear relation with the outcome by categorizing the variables into quartiles and determining whether they revealed a linear trend. None of the continuous variables showed a linear trend and all values were therefore grouped and included as categorical variables.

“Maternal age” and “number of siblings” were inter-correlated, and so were “health facility within the village” and “distance to the nearest health care facility”, as well as “region” and “maternal ethnicity”. We therefore only included the exposure which could be targeted in interventions or where the classification was subject to the least data collection error/inaccuracy. Thus, we included; number of siblings, health facility within the village, and region in the multivariate analyses. Furthermore, place of birth and skilled attendance was only available among children registered before birth and was therefore not included in the multivariate analyses.

Guinea-Bissau had two national OPV campaigns in 2002 and two in 2004. We split the observation time at the OPV campaigns and season to assess whether there were differences in mortality before and after the campaigns, adjusted for season and age.

Vaccination coverage was not included as a risk factor in the survival analyses as the vaccination status of dead children is uncertain especially in study settings with biannual visits [16]. We therefore only looked at overall vaccination coverage in the cohorts among the survivors, assuming that these children were representative of the cohort. We assessed difference in coverage from 1992–3 to 2002–3 using binomial regression.

We estimated the potential contribution of the change in coverage of MV and DTP vaccine on mortality. We based the calculations on recent meta-analyses of the effect of vaccines on all-cause mortality (16,17). For MV and DTP with/after MV we used the estimates reported in a recent independent WHO commissioned review. We assumed that having MV as the most recent vaccine was associated with 46% (35–55%) lower mortality—a difference which was more pronounced for girls (79%) than for boys (13%) [17]. Contrarily, MV and DTP vaccine co-administered was associated with 2.3 (1.6–3.4) times higher mortality compared with MV-only [17]. As argued elsewhere, the WHO commissioned review of DTP includes studies with methodological problems due to frailty and survival bias (17). We have therefore used the estimate for DTP as most recent vaccine, from a review of studies which document vaccination status and have prospective follow-up. In this review of DTP studies, DTP was associated with 2.00 (1.50–2.67) fold higher mortality compared with children who were not DTP vaccinated [18].

Finally, we assessed the impact of vaccination status during the second year of life on survival after infancy, comparing survival from first date of seen card between 12 and 23 months of age and six months onwards according to most recent vaccine: MV, DTP with or after MV, DTP only (no MV) or other/no vaccine, in a Cox regression, using age as the underlying time scale and stratifying by cohort.

All analyses were performed in STATA version 11.2 (StataCorp, College Station, TX) and adjusted for cluster sampling using robust variance estimates.

### Ethical considerations

The collection of data by the mobile teams has been going on in the current form since 1990 at the request of the Ministry of Health in Guinea-Bissau and UNICEF to determine child mortality levels in rural parts of Guinea-Bissau. The present study was conducted within the auspices of the OPTIMUNISE project, sponsored by the EU-FP7 programme. Bandim Health Project, as part of the National Institute of Health in Guinea-Bissau was a co-applicant for the OPTIMUNISE project; in Danish law register-based studies do not require separate ethical approval. Hence, no separate ethical permissions were sought for the specific analyses presented in this paper. Women of reproductive were registered after verbally consenting on behalf of themselves and their children under five at the time of registration. Written consent was not required by the ethics committees and was therefore not obtained. Only women who consented were registered and continuously followed. The ongoing data collection in its current form, has been approved by the ethical commission of Guinea-Bissau and Denmark. The present study was based on data collected years before the data extraction and analysis and to allow data cleaning by checking missing data and outliers on the original forms, data was available in non-anonymised form to the main author. The authors have all participated actively in supervision, data management and data analysis of the routine data collection by the mobile teams.

## Results

In 1992–1993, 3706 live born children were followed; in 2002–3 it was 4526 children. The overall infant mortality declined significantly from 148 (95%CI:135–162) per 1000 person years in 1992–3 to 124 (114–136) per 1000 person years in 2002–3 (Table 1), the hazard ratio (HR) being 0.88 (0.77–0.99). This was not a uniform decline as the mortality rates (MR) for 2002–3 compared with 1992–3 did not change for children aged 1.5–8 months of age (HR = 0.99;0.81–1.21), among neither boys nor girls (Table 1). The decline was largest among children 9–11 months of age (HR = 0.69;0.54–0.90), mainly due to a mortality reduction among girls in this age group (HR = 0.56;0.37–0.83) (Table 1). Mortality among neonatal girls also tended to be lower in 2002–3 than in 1992–3 (HR = 0.73;0.51–1.05) (S1 Table).

**Table 1:** Crude infant mortality rates in the 1992–3 and 2002–3 birth cohorts for all children and by age groups—Overall and by sex.

	Cohort 1992–3			Cohort 2002–3			Hazard ratio 2002–3 vs 1992–3 (95% CI)*
	N	Deaths/PYRS^a^	Mortality rate (per 1000 PYRS^a^) (95% CI)	N	Deaths/PYRS^a^	Mortality rate per 1000 PYRS^a^ (95% CI)	
**All children**	3706	471/3185	148 (135–162)	4526	470/3779	124 (114–136)	0.88 (0.77–0.99)
**0–45 days**	3360	177/381	464 (401–538)	3722	165/411	402 (345–468)	0.90 (0.72–1.11)
**1.5–8 months**	3461	171/1767	97 (83–112)	4176	199/2081	96 (83–110)	0.99 (0.81–1.21)
**9–11 months**	3266	123/1037	119 (99–142)	4075	106/1287	82 (68–100)	0.69 (0.54–0.90)
**Boys**^b^							
**All boys**	1899	256/1629	157 (139–178)	2304	280/1909	147 (130–165)	0.97 (0.82–1.15)^c^
**0–45 days**	1719	97/194	500 (410–611)	1907	101/209	484 (398–588)	1.00 (0.75–1.32)^d^
**1.5–8 months**	1772	93/907	103 (84–126)	2111	113/1054	107 (89–129)	1.05 (0.80–1.38) ^e^
**9–11 months**	1670	66/529	125 (98–159)	2052	66/646	102 (80–130)	0.82 (0.58–1.15)^f^
**Girls**^b^							
**All girls**	1803	212/1556	136 (119–156)	2221	189/1870	101 (88–117)	0.77 (0.64–0.94)^c^
**0–45 days**	1637	78/187	417 (334–520)	1814	64/202	317 (248–405)	0.79 (0.57–1.10)^d^
**1.5–8 months**	1687	77/860	90 (72–112)	2064	85/1027	83 (67–102)	0.93 (0.68–1.26)^e^
**9–11 months**	1596	57/508	112 (86–145)	2023	40/641	62 (46–85)	0.56 (0.37–0.83)^f^

*Estimated in a Cox proportional hazards model with age as underlying timescale; 95%CI adjusted for cluster sampling using robust variance estimates

^a^: PYRS: Person-years

^b^: Sex missing for 4 children in 1992–3 and for 1 child in 2002–3

^c^: p = 0.10 for interaction between cohort and sex

^d^: p = 0.30 for interaction between cohort and sex

^e^: p = 0.56 for interaction between cohort and sex

^f^: p = 0.15 for interaction between cohort and sex

Univariate and multivariate risk factor analyses are presented in Table 2. Being twin, being firstborn and previous loss of child were associated with higher MR in both cohorts. The MR did not differ according to region, season of birth and maternal schooling. Having no health center in the village was associated with higher mortality in 1992–3. In 2002–3, long distance to a hospital was associated with higher MR. The distribution of risk factors did not change from 1992–3 to 2002–3 except for previous loss of child, which was lower in 2002–3 (Table 2). Three quarters of the children in both cohorts were born at home, only 13 and 15% percent of the mothers had attended school and the distributions in maternal age did not differ by cohort.

**Table 2:** Distribution (frequencies) and association of potential risk factors with infant mortality in the 1992–3 and 2002–3 birth cohorts (univariate and multivariate analyses).

Risk factor	1992–3 cohort					2002–3 cohort					
	N	%	Mortality Rate pr. 1000 PYRS^a^	Hazard ratio (95%CI)*	Adjusted Hazard ratio (95%CI)*	N	%	Mortality Rate pr. 1000 PYRS^a^	Hazard ratio (95%CI)*	Adjusted Hazard ratio (95%CI)*	Interaction with cohort (p-value) Univariate/ Multivariate
**Sex of child**^b^				[p = 0.11]	[p = 0.44]				[p<0.01]	[p<0.01]	0.11 / 0.08
Boy	1899	51%	157 (139–177)	1.00 (ref)	1.00 (ref)	2304	51%	147 (130–165)	1.00 (ref)	1.00 (ref)	
Girl	1803	49%	136 (119–156)	0.86 (0.72–1.04)	0.92 (0.75–1.13)	2221	49%	101 (88–116)	0.69 (0.58–0.83)	0.72 (0.59–0.87)	
**Region of birth**				[p = 0.08]	[p = 0.73]				[p = 0.58]	[p = 0.73]	0.46 / 0.67
Oio	802	22%	167 (139–201)	1.00 (ref)	1.00 (ref)	945	21%	115 (93–141)	1.00 (ref)	1.00 (ref)	
Biombo	929	25%	157 (132–187)	0.94 (0.73–1.22)	0.83 (0.59–1.17)	1055	23%	133 (111–159)	1.19 (0.91–1.57)	1.07 (0.79–1.47)	
Gabu	761	21%	134 (109–165)	0.81 (0.61–1.06)	0.87 (0.60–1.27)	934	21%	114 (93–139)	0.98 (0.74–1.31)	1.00 (0.73–1.36)	
Cacheu	534	14%	132 (113–174)	0.81 (0.59–1.11)	0.80 (0.56–1.14)	799	18%	125 (100–156)	1.12 (0.83–1.52)	1.04 (0.75–1.45)	
Bafata	680	18%	140 (113–174)	0.83 (0.63–1.11)	0.80 (0.56–1.14)	793	18%	137 (111–168)	1.19 (0.89–1.59)	1.22 (0.89–1.67)	
**Season of birth**				[p = 0.42]	[p = 0.21]				[p = 0.72]	[p = 0.60]	0.41/ 0.22
Rainy season	1665	45%	153 (134–175)	1.00 (ref)	1.00 (ref)	1965	43%	123 (107–142)	1.00 (ref)	1.00 (ref)	
Dry season	2041	55%	144 (127–162)	0.93 (0.78–1.11)	0.90 (0.75–1.07)	2561	57%	125 (111–141)	1.03 (0.86–1.24)	1.05 (0.87–1.28)	
**Maternal age at child birth**				[p = 0.24]					[p = 0.81]		0.26/ NA
<20 years	805	22%	185 (155–221)	1.00 (ref)		988	22%	150 (125–181)	1.00 (ref)		
20–24 years	942	25%	120 (99–146)	0.65 (0.50–0.85)		1347	30%	108 (91–129)	0.71 (0.55–0.91)		
25–29 years	933	25%	153 (128–182)	0.83 (0.64–1.06)		1021	23%	101 (82–125)	0.65 (0.49–0.86)		
30–34 years	614	17%	139 (111–174)	0.76 (0.57–1.01)		662	15%	146 (118–182)	0.93 (0.70–1.23)		
35+ years	412	11%	143 (109–189)	0.78 (0.56–1.09)		508	11%	138 (107–177)	0.88 (0.65–1.20)		
**Ethnicity of mother**^d^				[p = 0.20]					[p = 0.08]		0.07/ NA
Fula	975	26%	150 (126–179)	1.00 (ref)		849	19%	111 (92–133)	1.00 (ref)		
Balanta	681	18%	183 (151–221)	1.21 (0.93–1.57)		1181	26%	107 (85–134)	0.99 (0.74–1.32)		
Mandinga	744	20%	125 (100–155)	0.83 (0.63–1.10)		974	22%	151 (127–180)	1.38 (1.07–1.78)		
Manjaco	236	6%	106 (70–161)	0.72 (0.46–1.14)		350	8%	99 (68–143)	0.94 (0.62–1.42)		
Pepel	789	21%	153 (126–185)	1.02 (0.79–1.32)		871	19%	142 (117–172)	1.33 (1.02–1.74)		
Other	281	8%	141 (100–198)	0.95 (0.65–1.39)		292	6%	118 (81–171)	1.09 (0.72–1.64)		
**Maternal schooling**^e^				[p = 0.74]	[p = 0.84]				[p = 0.79]	[p = 0.85]	0.70/0.97
Never went to school	3198	87%	148 (135–164)	1.00 (ref)	1.00 (ref)	3793	85%	126 (114–139)	1.00 (ref)	1.00 (ref)	
Went to school	466	13%	141 (109–183)	0.95 (0.72–1.26)	1.04 (0.73–1.47)	691	15%	118 (93–151)	0.96 (0.74–1.25)	0.97 (0.74–1.28)	
**Health facility within the village?**				[p = 0.01]	[p = 0.04]				[p = 0.51]	[p = 0.98]	0.16/0.22
Yes	1893	51%	130 (113–148)	1.00 (ref)	1.00 (ref)	2251	50%	121 (106–137)	1.00 (ref)	1.00 (ref)	
No	1813	49%	167 (148–189)	1.28 (1.07–1.53)	1.29 (1.01–1.66)	2275	50%	128 (113–145)	1.06 (0.89–1.27)	1.00 (0.81–1.23)	
**Distance to nearest health facility (any kind)**				[p = 0.04]					[p = 0.88]		0.51/NA
<1 km	1893	51%	130 (113–148)	1.00 (ref)		2275	50%	121 (106–137)	1.00 (ref)		
1–4 km	835	23%	176 (147–210)	1.35 (1.08–1.68)		979	22%	135 (112–163)	1.13 (0.90–1.41)		
5–9 km	662	18%	152 (124–188)	1.17 (0.91–1.50)		827	18%	123 (100–152)	1.03 (0.80–1.31)		
10+ km	316	9%	175 (131–233)	1.33 (0.97–1.83)		445	10%	121 (90–162)	1.00 (0.72–1.37)		
**Distance to nearest hospital**				[p = 0.23]	[p = 0.50]				[p = 0.01]	[p = 0.03]	0.64/0.55
<10 km	614	17%	141 (112–177)	1.00 (ref)	1.00 (ref)	686	15%	96 (74–126)	1.00 (ref)	1.00 (ref)	
10–20 km	666	18%	142 (114–176)	1.01 (0.74–1.38)	0.96 (0.69–1.32)	967	21%	120 (98–146)	1.23 (0.89–1.71)	1.36 (0.96–1.92)	
21–29 km	685	18%	129 (104–162)	0.92 (0.67–1.26)	0.85 (0.59–1.23)	813	18%	108 (86–136)	1.11 (0.78–1.57)	1.15 (0.79–1.68)	
30+ km	1741	47%	160 (141–182)	1.14 (0.88–1.48)	1.13 (0.87–1.46)	2060	46%	142 (126–162)	1.48 (1.11–1.98)	1.54 (1.13–2.11)	
**Place of giving birth**^c^^,^^f^				[p = 0.78]					[0.13]		0.23/NA
At hospital/health centre	823	27%	143 (120–171)	1.00 (ref)		800	26%	149 (123–180)	1.00 (ref)		
At home	2198	73%	146 (131–162)	0.97 (0.78–1.21)		2308	74%	125 (111–141)	0.84 (0.67–1.05)		
**Born with skilled attendance?**^c^^,^^g^				[p = 0.35]					[p = 0.10]		0.08/NA
Yes	1085	37%	136 (115–162)	1.00 (ref)		1152	38%	145 (123–170)	1.00 (ref)		
No	1814	63%	151 (133–171)	1.11 (0.90–1.36)		1852	62%	119 (104–137)	0.83 (0.66–1.03)		
**Was the child a twin?**^h^				[p<0.01]	[p<0.01]				[p<0.01]	[p<0.01]	0.46/0.41
No	3602	97%	143 (131–157)	1.00 (ref)	1.00 (ref)	4367	96%	118 (107–129)	1.00 (ref)	1.00 (ref)	
Yes	102	3%	333 (227–489)	2.23 (1.50–3.30)	2.51 (1.58–3.98)	159	4%	334 (244–457)	2.70 (1.94–3.75)	2.96 (2.09–4.21)	
**Number of older siblings for the child**^i^				[p = 0.01]	[p<0.01]				[p = 0.39]	[p<0.01]	0.06/0.08
0	506	15%	224 (182–274)	1.00 (ref)	1.00 (ref)	671	17%	159 (129–196)	1.00 (ref)	1.00 (ref)	
1–2	1118	34%	133 (112–158)	0.60 (0.46–0.78)	0.52 (0.39–0.71)	1423	36%	123 (104–145)	0.81 (0.62–1.06)	0.69 (0.52–0.91)	
3–4	486	15%	151 (118–193)	0.67 (0.49–0.93)	0.57 (0.39–0.82)	590	15%	91 (68–122)	0.59 (0.41–0.84)	0.47 (0.32–0.69)	
≥5	1157	35%	135 (114–160)	0.61 (0.47–0.80)	0.46 (0.31–0.68)	1289	32%	137 (117–160)	0.87 (0.67–1.13)	0.58 (0.42–0.80)	
**Previously lost a child**				[p = 0.52]	[p = 0.05]				[p<0.01]	[p<0.01]	0.10/0.12
No	1754	47%	142 (125–163)	1.00 (ref)	1.00 (ref)	2550	56%	108 (95–123)	1.00 (ref)	1.00 (ref)	
Yes	1952	53%	153 (135–172)	1.06 (0.88–1.27)	1.33 (1.01–1.77)	1976	44%	145 (128–164)	1.31 (1.10–1.58)	1.56 (1.23–2.00)	

*Estimated in a Cox proportional hazards model with age as underlying timescale; 95%CI adjusted for cluster sampling using robust variance estimates

^a^: PYRS: Person-years

^b^: Sex missing for 4 children in 1992–3 and for 1 child in 2002–3

^c^: Only for children registered before birth

^d^: Ethnicity missing for 9 mothers in 2002–3

^e^: Education missing for 42 mothers in both 1992–3 and in 2002–3

^f^: Place of giving birth missing for 83 infants in 1992–3 and for 33 in 2002–3

^g^: Skilled attendance at birth missing for 205 infants in 1992–3 and for 137 in 2002–3

^h^: Twin status missing for 2 infants in 1992–3

^i^: Number of older siblings missing for 439 infants in 1992–3 and for 553 in 2002–3

Stratified by age group, the main factor associated with mortality decline was sex (S2 Table): In 2002–3, girls aged 0–45 days and 9–11 months had significantly lower MR compared with boys, while mortality of boys and girls did not differ markedly in any age group in 1992–3. We found that distance to nearest hospital, being firstborn, being twin and previous loss of child mainly were risk factors in younger infants. We saw no statistical interactions between the examined risk factors and sex (data not shown) and between the examined risk factor and cohort (Table 2, S2 Table). Four OPV campaigns took place during follow-up time for the 2002–3 cohort (S3 Table). After splitting the observation time by OPV campaign dates, 26% (993/3782) of the time was spent as exposed to campaign-OPV. Adjusted for age and season, mortality tended to be lower after the OPV campaigns (HR = 0.90;0.69–1.17) due to a lower mortality for boys (HR 0.81;0.58–1.14) (data not shown).

**Fig 1:**
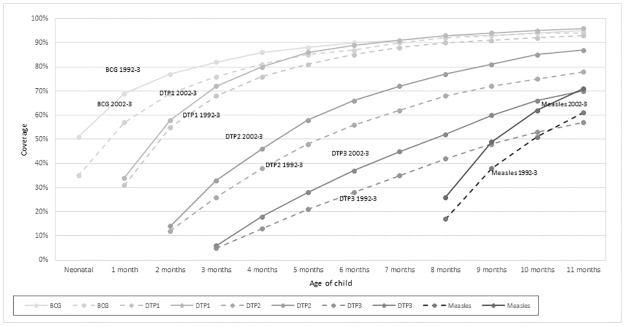
Vaccination coverage with BCG and DTP in the 1992–3 and 2002–3 birth cohorts among children with a seen vaccination card within 12 months of the assessment age.

We inspected vaccination cards of more than 2000 children in both 1992–3 and 2002–3 and calculated vaccination coverage by age (months) (Fig 1). BCG coverage was higher in 1992–3 compared with 2002–3, but equaled out at 7 months of age. The DTP coverage was higher in all relevant age groups in 2002–3, as was the MV coverage among 9–11 month children in 2002–3 compared with 1992–3 (Fig 1), p<0.001. There was no difference in vaccination coverage between boys and girls (data not shown). Significantly more children had MV as their most recent vaccine in 2002–3 (41%) compared with 1992–3 (30%) (RR = 1.38;1.28–1.50) (Table 3). After infancy, the HR of death was higher among children with DTP> = MV (HR = 1.44 (0.97–2.15); other vaccines/no vaccine compared with children who had MV as last vaccine was also associated with increased mortality, the HR being 2.58 (1.54–4.33). Not having MV-only as last vaccine tended to be worse for girls (HR = 1.63 (1.14–2.32)) than for boys (HR = 1.24 (0.85–1.81)) (Table 4).

**Table 3:** Relative risk (RR) of DTP, MV and DTP, MV-only or other/no vaccines as most recent vaccination by 9, 10 and 11 months of age by cohort.

	DTP %(n)			DTP after MV or DTP and MV together %(n)			MV-only%(n)			Other^a^ or no vaccines %(n)	
	1992–3	2002–3	RR (95%CI)*	1992–3	2002–3	RR (95%CI)*	1992–3	2002–3	RR (95%CI)*	1992–3	2002–3
**9 months**	53% (1120)	45% (1175)	0.84 (0.80–0.89)	18% (387)	19% (502)	1.04 (0.92–1.17)	19% (404)	29% (755)	1.50 (1.35–1.67)	8% (196)	7% (190)
1992–3 n = 2107											
2002–3 n = 2622											
**10 months**	42% (864)	33% (840)	0.79 (0.73–0.85)	25% (514)	25% (633)	1.00 (0.90–1.10)	26% (531)	36% (927)	1.41 (1.29–1.54)	7% (169)	4% (171)
1992–3 n = 2078											
2002–3 n = 2571											
**11 months**	33% (668)	25% (631)	0.76 (0.69–0.83)	31% (623)	28% (712)	0.92 (0.84–1.01)	30% (606)	41% (1042)	1.38 (1.28–1.50)	6% (145)	3% (151)
1992–3 n = 2042											
2002–3 n = 2536											

^a^: Includes BCG only or OPV-only or BCG and OPV only

*RR assessed in binomial regression model with a log-link function; 95% CI adjusted for cluster sampling using robust variance estimates

**Table 4:** Mortality (Hazard ratios (95%CI)) 6 months after seeing a vaccination card between 12 and 23 months according to having MV, DTP, DTP> = MV or other or no vaccine as most recent vaccination; overall and by sex^a^.

	Overall		Boys		Girls	
	Deaths / person years	HR (95%CI)*	Deaths / person years	HR (95%CI)	Deaths / person years	HR (95%CI)*
**MV-only**	46/775	1.00 (ref)	24/411	1.00 (ref)	22/364	1.00 (ref)
**DTP**	34/611	0.92 (0.59–1.43)	14/296	0.77 (0.40–1.50)	20/315	1.05 (0.57–1.93)
**DTP> = MV**	53/614	1.44 (0.97–2.15)	24/301	1.38 (0.78–2.44)	29/313	1.52 (0.87–2.65)
**Other or no vaccine**^b^	21/132	2.58 (1.54–4.33)	9/71	1.97 (0.91–4.25)	12/61	3.27 (1.61–6.64)

^**a**^Children were followed from first date of seeing the vaccination card between 12 and 23 months and six months onward.

*HR: Hazard Ratio estimated in a Cox proportional hazards model with age as underlying timescale and stratified by cohort; 95%CI adjusted for cluster sampling using robust variance estimates

^b^Other vaccine: BCG or OPV or both

Based on the effect estimates from the recent WHO review of the non-specific effects of vaccines [17], the increase in the proportion having MV as the most recent vaccine and the lower proportion having DTP or co-administered DTP and MV as the most recent vaccine ‘explained’ a 6% decline in overall mortality between 1992–3 and 2002–3, equivalent to 19% of the mortality reduction from 1992–3 to 2002–3. This was 8% (18% of the mortality reduction) for girls and 2% (11%) for boys. Among children aged 1–8 months (“DTP-age”), the higher DTP coverage coverage accounted for an estimated 2% increased mortality under the assumption that DTP vaccinated children had double the mortality of DTP unvaccinated children [18] (S4 Table).

## Discussion

Infant mortality declined significantly from 1992–3 to 2002–3. The change was heterogeneous with respect to sex and age group, being strongest for older girls. Distribution of risk factors did not change from 1992–3 to 2002–3. Being twin, previous loss of child and being the first born child were associated with higher MR in both 1992–3 and 2002–3. Vaccination coverage was higher in 2002–3 except for BCG vaccination. A higher proportion of children had MV-only as most recent vaccine in 2002–3 compared with 1992–3 and a lower proportion had DTP and MV out of sequence. The higher proportion with MV-only as last vaccine in 2002–3 may have contributed to the stronger mortality decline for girls.

The main strength of this study is the large number of children prospectively surveyed by BHP. The longstanding registration of pregnancies ensures accurate information on early neonatal mortality, which in situations without reconciliation between pregnancies and child records are likely to be underreported [19]. Due to the random selection of clusters, we believe that the sampled children are representative of the rural population of Guinea-Bissau. Furthermore, we were able to investigate several risk factors including both maternal and infant risk factors assessed prior to the beginning of follow up, and assess their influence on infant mortality without response bias.

Nonetheless, some limitations must be considered. First, we investigate many potential risk factors which increases the risk of chance findings. In spite of the many factors included, there were many factors which we did not have information about: We were unable to include factors related to antenatal care as information on antenatal care was only collected in 2002–3. Antenatal care has been associated with lower infant mortality in Ethiopia [20], in South Africa [12] and in rural India [21]. Information on maternal nutritional status during pregnancy was not available. Virtually all children (>95%) in both cohorts were still breastfeeding at one year of age. Changes in breastfeeding practices are therefore unlikely to explain the decline in mortality. Similarly, paternal factors as well as ability to pay for antenatal and delivery services, and maternal health are not taken into account. HIV has been suggested to underlie a large share of the infant mortality burden in Africa through transmission from mother to child [22]. Although we are unaware of the HIV status of our study population, a HIV prevalence survey reported the prevalence in children <5 years to be 0.2% in 2010 [23]. Since we have registered number of previous births in the beginning of the study, and not their exact year, we were not able to look at birth spacing as a risk factor for infant mortality, although insufficient spacing is known to influence the risk of infant mortality [24, 25]. We were only able to look at vaccination coverage overall and not to link vaccination status of the individual child to mortality during infancy, as we would otherwise risk introducing survival bias [16]. Furthermore, we were only able to assess vaccination status among children with a vaccination card seen during the home visits; only slightly more than half of the children had their vaccination card inspected.

Infant mortality has declined in most low-income countries from the early 1990’s up until today [3]. West Africa has experienced some of the greatest reductions with falls of on average 25 deaths/1000 births from 1990 until 2000 [26], similar to the numbers in the present study. Unlike some African regions, where the HIV epidemic has resulted in a recent increase in child mortality, West Africa has maintained its steady decline [26, 27].

The most important risk factors for infant death in our study were: firstborn, twin status and previous loss of child—especially among the youngest children. The importance of these factors have been reported in several other studies of infant mortality [25, 26, 28, 29] and did not change markedly over time in the present study. A study from nearby Senegal has suggested an effect of season on mortality [24]. We did not see an effect of season of birth in the present study. However, this could be because we only investigate infant mortality and season of birth—the effect of season seems to be more pronounced in the older age groups [24, 27].

Higher level of maternal education has been associated with a more active health seeking behaviour for childhood illnesses [30]. As the level of education is generally low in Guinea-Bissau and the health centres where mothers would seek care, often lack essential drugs and other necessities, the influence of maternal education may be limited in this setting, and explain why we neither found any association with maternal education or a consistent effect of distance to the health centre.

Living close to a hospital (<10 km) was associated with lower risk of infant mortality in 2002–3 –a change from 1992–3 where distance to hospital was not associated with infant mortality. While this could be due to improved treatment regimens in the hospitals or better supplies of essential medicines, we cannot rule out that this could be a chance finding.

Although we found some factors to differ according to mortality risk from 1992–3 to 2002–3 (e.g. distance to health centre/hospital) most did not and the distributions of risk factors did not change. Hence, none of the investigated risk factors appear to explain the observed mortality decline. We therefore looked for other explanations of the mortality decline. Although many factors may explain the decline in infant mortality, we looked in particular to the effects of changes in childhood interventions (S3 Table). Childhood interventions have increased in frequency and coverage over time and may have a major effect on child mortality [31]. Bed net campaigns became more frequent from 2000 and onwards, however, bed net use at first visit after birth was much lower in 2002–3 (29%) compared with 1992–3 (68%), and is therefore unlikely to explain the mortality decline.

Most notably, we saw that the infant mortality declined relatively more among older girls (9–11 months). An assessment of global sex ratios of infant mortality found girls to have an increasing survival advantage as total infant mortality declined [32]. Furthermore, sentinel malaria surveillance from urban Guinea-Bissau shows that the malaria prevalence dropped markedly from 1995–2003 in Bissau city [33] and thus, this decline may have contributed to the decline in mortality. However, the decline in malaria prevalence is not likely to explain the sex-differential decline in mortality among older children or the age-differential decline between before and after 9 months of age.

Our study offers an alternative explanation as the decline differed both by sex and age, being stronger for girls than for boys and more pronounced after 9 months of age. In the present study, MV coverage between 9 and 11 months was higher and more children had MV as last vaccine in 2002–3 compared with 1992–3. Furthermore, we found mortality between 9–11 months of age to be lower for girls from 1992–3 to 2002–3 relative to boys, in line with studies of non-specific effects of MV showing a more beneficial effect for girls [34, 35]. The DTP vaccine has been associated with negative non-specific effects on mortality in girls [36]. The coverage of DTP1, DTP2 and DTP3 vaccination was higher at all ages in 2002–3 compared with 1992–3 though the difference was smaller than for MV. Using the meta-estimates from WHO, we estimated the contribution of increased coverage of MV to be 6% on overall mortality, roughly accounting for 19% of the mortality decline observed among 9–11 month old infants from 1992–3 to 2002–3. This decline was 8% for girls and 2% for boys. The actual effects of MV may be higher. Thus, the increase in MV coverage may contribute to the observed decline in infant mortality among girls, though it cannot explain the entire decline.

In contrast to the oldest infants, we saw higher or similar HRs in 2002–3 and 1992–3 from 1.5 to 8 months of age where DTP vaccination is likely to be the most recent vaccine. This is consistent with our calculations showing that DTP may account for a mortality increase of 2%. Mortality tended to be higher after infancy in girls with DTP or DTP> = MV as the most recent vaccine six months after vaccine assessment date. Although we did not detect a significant overall effect of the OPV campaigns after splitting the observation time, we did see a non-significant decrease in mortality among boys exposed to OPV campaign compared with boys who were not exposed to OPV campaign. This is in line with a previous RCT and campaign studies showing that OPV has strong beneficial effects and that these effects are somewhat stronger for boys than girls [31,37] The potential beneficial contribution of OPV campaigns is likely to have been masked by the increase in DTP coverage, and vice versa the OPV campaigns may have averted some of the detrimental effects of DTP among 1–8 month old children.

Hence, the increase in childhood interventions including routine vaccinations and vaccination campaigns are likely to have contributed to the observed mortality decline.

### Conclusions

Infant mortality declined from 1992–3 to 2002–3. Risk factors did not change over time. The decline was mainly seen among the oldest infants and among girls. The increased coverage of MV may have contributed to the decline. More attention should be directed towards understanding sex-differential mortality changes.

## Supporting information

S1 Table Hazard ratios in the 1992–3 and 2002–3 birth cohorts in monthly intervals—Overall and by sex. (DOCX)

S2 Table Risk factors for infant mortality in the 1992–3 and 2002–3 birth cohorts (multivariate analysis)—by age groups. (DOCX)

S3 Table Child health interventions provided to infants born in 1992–3 and 2002–3 in Guinea-Bissau. (DOCX)

S4 Table Calculation of vaccine contribution to mortality (example for MV as last vaccine). (DOCX)
