# Supplementary material for: Analysis of risk factors for infant mortality in the 1992-3 and 2002-3 birth cohorts in rural Guinea-Bissau
Source: PLoS One. 2017 May 18;12(5):e0177984. doi: 10.1371/journal.pone.0177984 (PMC5436893; doi:10.1371/journal.pone.0177984)
Supplement: S1 Table — (DOCX) [file pone.0177984.s001.docx]

**S1 Table. Hazard ratios in the 1992-3 and 2002-3 birth cohorts in monthly intervals – Overall and by sex**

|  | **All children** | | | **Boys** | | | **Girls** | | |
| --- | --- | --- | --- | --- | --- | --- | --- | --- | --- |
|  | **Cohort 1992-3 (n)** | **Cohort 2002-3 (n)** | **Hazard Ratio 2002-3 vs 1992-3 (95% CI)** | **Cohort 1992-3 (n)** | **Cohort 2002-3 (n)** | **Hazard Ratio 2002-3 vs 1992-3 (95% CI)** | **Cohort 1992-3 (n)** | **Cohort 2002-3 (n)** | **Hazard Ratio 2002-3 vs 1992-3 (95% CI)** |
| **Neonatal** | 3295 | 3568 | 0.87 (0.70-1.09) | 1683 | 1831 | 0.99 (0.75-1.31) | 1608 | 1736 | 0.73 (0.51-1.05) |
| **1 month** | 3250 | 3644 | 1.25 (0.71-2.19) | 1656 | 1848 | 1.47 (0.61-3.54) | 1592 | 1795 | 1.11 (0.53-2.31) |
| **2 months** | 3281 | 3767 | 1.14 (0.67-1.95) | 1676 | 1916 | 1.17 (0.57-2.40) | 1603 | 1805 | 1.23 (0.55-2.77) |
| **3 months** | 3277 | 3838 | 1.08 (0.66-1.77) | 1682 | 1952 | 0.87 (0.46-1.64) | 1594 | 1885 | 1.53 (0.67-3.45) |
| **4 months** | 3279 | 4895 | 1.06 (0.62-1.82) | 1681 | 1979 | 1.81 (0.82-4.01) | 1598 | 1915 | 0.62 (0.28-1.34) |
| **5 months** | 3272 | 3931 | 0.80 (0.44-1.46) | 1683 | 1987 | 0.66 (0.29-1.49) | 1589 | 1943 | 1.01 (0.42-2.44) |
| **6 months** | 3279 | 3951 | 0.80 (0.49-1.33) | 1687 | 1991 | 0.85 (0.41-1.73) | 1592 | 1959 | 0.76 (0.38-1.55) |
| **7 months** | 3262 | 3955 | 1.02 (0.60-1.73) | 1674 | 1994 | 0.95 (0.48-1.86) | 1588 | 1960 | 1.08 (0.45-2.56) |
| **8 months** | 3244 | 3963 | 0.85 (0.52-1.39) | 1660 | 1995 | 0.94 (0.48-1.84) | 1584 | 1968 | 0.75 (0.36-1.56) |
| **9 months** | 3213 | 3954 | 0.46 (0.27-0.79) | 1639 | 1991 | 0.64 (0.32-1.29) | 1574 | 1963 | 0.29 (0.12-0.70) |
| **10 months** | 3175 | 3977 | 0.70 (0.43-1.16) | 1620 | 1998 | 0.77 (0.40-1.49) | 1555 | 1979 | 0.63 (0.30-1.35) |
| **11 months** | 3145 | 3969 | 0.84 (0.52-1.38) | 1600 | 1990 | 1.09 (0.58-2.04) | 1545 | 1979 | 0.56 (0.25-1.26) |
