# Supplementary material for: Analysis of risk factors for infant mortality in the 1992-3 and 2002-3 birth cohorts in rural Guinea-Bissau
Source: PLoS One. 2017 May 18;12(5):e0177984. doi: 10.1371/journal.pone.0177984 (PMC5436893; doi:10.1371/journal.pone.0177984)
Supplement: S2 Table — (DOCX) [file pone.0177984.s002.docx]

**S2 Table. Risk factors for infant mortality in the 1992-3 and 2002-3 birth cohorts (multivariate analysis) - by age groups**

|  | **0-45 days** | | | **1.5 – 8 months** | | | **9-11 months** | | |
| --- | --- | --- | --- | --- | --- | --- | --- | --- | --- |
| **Potential risk factor** | **1992-3 cohort** | **2002-3 cohort** | **Interaction with cohort** | **1992-3 cohort** | **2002-3 cohort** | **Interaction with cohort** | **1992-3 cohort** | **2002-3 cohort** | **Interaction with cohort** |
|  | **Hazard Ratio (95%CI)*** | **Hazard Ratio (95%CI)*** |  | **Hazard Ratio (95%CI)*** | **Hazard Ratio (95%CI)*** |  | **Hazard Ratio (95%CI)*** | **Hazard Ratio (95%CI)*** |  |
| **Sex of child** | P=0.55 | P=0.04 | P=0.33 | P=0.83 | P=0.11 | P=0.37 | P=0.45 | P=0.04 | P=0.35 |
| Boy | 1.00 (ref) | 1.00 (ref) |  | 1.00 (ref) | 1.00 (ref) |  | 1.00 (ref) | 1.00 (ref) |  |
| Girl | 0.90 (0.66-1.25) | 0.70 (0.50-0.97) |  | 0.97 (0.69-1.34) | 0.78 (0.57-1.06) |  | 0.87 (0.59-1.26) | 0.64 (0.42-0.97) |  |
| **Region of birth** | P=0.02 | P=0.14 | P=0.46 | P=0.70 | P=0.83 | P=0.94 | P=0.05 | P=0.12 | P=0.99 |
| Oio | 1.00 (ref) | 1.00 (ref) |  | 1.00 (ref) | 1.00 (ref) |  | 1.00 (ref) | 1.00 (ref) |  |
| Biombo | 0.40 (0.24-0.68) | 0.83 (0.47-1.45) |  | 1.05 (0.63-1.76) | 1.18 (0.70-1.96) |  | 1.48 (0.84-2.63) | 1.53 (0.81-2.86) |  |
| Gabu | 0.77 (0.47-1.26) | 1.03 (0.61-1.74) |  | 1.29 (0.77-2.17) | 1.26 (0.78-2.05) |  | 0.65 (0.34-1.25) | 0.66 (0.32-1.36) |  |
| Cacheu | 0.77 (0.45-1.31) | 1.22 (0.70-2.13) |  | 0.82 (0.44-1.54) | 1.14 (0.67-1.95) |  | 0.90 (0.45-1.80) | 0.92 (0.46-1.87) |  |
| Bafata | 0.77 (0.48-1.24) | 1.55 (0.93-2.58) |  | 0.98 (0.56-1.70) | 1.33 (0.80-2.22) |  | 0.68 (0.35-1.35) | 0.80 (0.38-1.70) |  |
| **Season of birth** | P=0.16 | P=0.67 | P=0.48 | P=0.21 | P=0.18 | P=0.98 | P=0.13 | P=0.50 | P=0.58 |
| Rainy season | 1.00 (ref) | 1.00 (ref) |  | 1.00 (ref) | 1.00 (ref) |  | 1.00 (ref) | 1.00 (ref) |  |
| Dry season | 0.80 (0.58-1.10) | 0.93 (0.67-1.29) |  | 1.24 (0.89-1.74) | 1.24 (0.91-1.69) |  | 0.75 (0.51-1.09) | 0.87 (0.57-1.31) |  |
| **Maternal schooling** | P=0.95 | P=0.12 | P=0.22 | P=0.36 | P=0.85 | P=0.52 | P=0.31 | P=0.15 | P=0.83 |
| Never attended school | 1.00 (ref) | 1.00 (ref) |  | 1.00 (ref) | 1.00 (ref) |  | 1.00 (ref) | 1.00 (ref) |  |
| Attended school | 0.98 (0.59-1.62) | 0.64 (0.37-1.13) |  | 0.77 (0.44-1.35) | 0.96 (0.62-1.49) |  | 1.32 (0.78-2.24) | 1.46 (0.88-2.43) |  |
| **Health facility within the village?^a^** | P=0.07 | P=0.99 | P=0.51 | P=0.33 | P=0.84 | P=0.40 | P=0.40 | P=0.87 | P=0.58 |
| Yes | 1.00 (ref) | 1.00 (ref) |  | 1.00 (ref) | 1.00 (ref) |  | 1.00 (ref) | 1.00 (ref) |  |
| No | 1.38 (0.97-1.96) | 1.00 (0.71-1.41) |  | 1.20 (0.83-2.30) | 0.97 (0.69-1.34) |  | 1.20 (0.79-1.83) | 0.96 (0.62-1.51) |  |
| **Distance to nearest hospital** | P=0.20 | P=0.03 | P=0.30 | P=0.33 | P=0.13 | P=0.59 | P=0.94 | P=0.74 | P=0.87 |
| <10 km | 1.00 (ref) | 1.00 (ref) |  | 1.00 (ref) | 1.00 (ref) |  | 1.00 (ref) | 1.00 (ref) |  |
| 10-20 km | 0.85 (0.49-1.48) | 2.10 (1.14-3.86) |  | 1.07 (0.59-1.95) | 0.83 (0.47-1.47) |  | 1.03 (0.53-2.00) | 1.46 (0.72-2.97) |  |
| 21-29 km | 0.61 (0.34-1.08) | 1.22 (0.61-2.46) |  | 0.93 (0.50-1.75) | 1.21 (0.69-2.14) |  | 1.17 (0.60-2.30) | 1.13 (0.51-2.51) |  |
| 30+ km | 1.03 (0.66-1.61) | 2.00 (1.13-3.53) |  | 1.39 (0.84-2.30) | 1.38 (0.86-2.23) |  | 0.98 (0.56-1.72) | 1.22 (0.63-2.34) |  |
| **Was the child a twin?** | P<0.01 | P<0.01 | P=0.11 | P=0.03 | P=0.17 | P=0.80 | P=0.04 | P=0.23 | P=0.64 |
| No | 1.00 (ref) | 1.00 (ref) |  | 1.00 (ref) | 1.00 (ref) |  | 1.00 (ref) | 1.00 (ref) |  |
| Yes | 2.86 (1.51-5.41) | 5.22 (3.24-8.40) |  | 2.42 (1.11-5.30) | 1.66 (0.81-3.42) |  | 2.36 (1.02-5.47) | 1.75 (0.70-4.38) |  |
| **Number of older siblings^b^** | P<0.01 | P<0.01 | P=0.15 | P=0.15 | P=0.15 | P=0.54 | P=0.46 | P=0.53 | P=0.73 |
| 0 | 1.00 (ref) | 1.00 (ref) |  | 1.00 (ref) | 1.00 (ref) |  | 1.00 (ref) | 1.00 (ref) |  |
| 1-2 | 0.85 (0.49-1.48) | 0.48 (0.29-0.77) |  | 0.59 (0.37-0.95) | 0.77 (0.49-1.20) |  | 0.71 (0.38-1.32) | 1.27 (0.64-2.50) |  |
| 3-4 | 0.61 (0.34-1.08) | 0.34 (0.18-0.65) |  | 0.58 (0.31-1.08) | 0.53 (0.29-0.97) |  | 0.54 (0.24-1.20) | 0.76 (0.32-1.83) |  |
| ≥5 | 1.03 (0.66-1.61) | 0.39 (0.22-0.66) |  | 0.57 (0.31-1.03) | 0.59 (0.35-1.01) |  | 0.59 (0.28-1.23) | 1.06 (0.49-2.30) |  |
| **Previously lost a child** | P=0.05 | P<0.01 | P=0.11 | P=0.77 | P=0.06 | P=0.09 | P=0.04 | P=0.17 | P=0.98 |
| No | 1.00 (ref) | 1.00 (ref) |  | 1.00 (ref) | 1.00 (ref) |  | 1.00 (ref) | 1.00 (ref) |  |
| Yes | 1.55 (1.00-2.41) | 1.94 (1.26-2.99) |  | 0.93 (0.60-1.46) | 1.44 (0.98-2.12) |  | 1.72 (1.03-2.87) | 1.42 (0.86-2.34) |  |

*Estimated in a Cox proportional hazards model with age as underlying timescale; 95%CI adjusted for cluster sampling using robust variance estimates

a: Due to collinearity between having a health centre within the village and distance to the health centre only having a health centre in the village is included in the multivariate analyses

b: Due to collinearity between maternal age at birth and number of siblings, only number of siblings is included in the multivariate analyses.
