# Supplementary material for: Analysis of risk factors for infant mortality in the 1992-3 and 2002-3 birth cohorts in rural Guinea-Bissau
Source: PLoS One. 2017 May 18;12(5):e0177984. doi: 10.1371/journal.pone.0177984 (PMC5436893; doi:10.1371/journal.pone.0177984)
Supplement: S3 Table — (DOCX) [file pone.0177984.s003.docx]

**S3 Table. Child health interventions provided to infants born in 1992-3 and 2002-3 in Guinea-Bissau**

| **Child interventions** | **Target age groups** | **1992-93 Cohort** | **2002-03 Cohort** |
| --- | --- | --- | --- |
| National OPV campaign | 0-59 months | None | 4 campaigns (5-8 October 2002; 9-12 November 2002; 18-21 October 2004; 18-21 November 2004) |
| National Vitamin A supplementation (VAS) campaign | 6-59 months | None | 3 campaigns (9-12 November 2002; 15-19 November 2003; 18-21 November 2004) |
| Bednet distribution | Whole population | None | 1 (2003) |
| Bednet coverage (assessed from routine data) | 0-59 months | 68% | 29% |
| **Routine vaccination coverage** |  |  |  |
| BCG | 1 month / 6 months / 12 months | 51% / 90% / 95% | 35% / 87% / 94% |
| DTP1 | 2 months / 6 months / 12 months | 31% / 85% / 93% | 34% / 89% / 96% |
| DTP3 | 4 months / 6 months / 12 months | 5% / 28% / 57% | 6% / 37% / 70% |
| MV | 9 months / 12 months | 38% / 61% | 49% / 71% |
