# Supplementary material for: Analysis of risk factors for infant mortality in the 1992-3 and 2002-3 birth cohorts in rural Guinea-Bissau
Source: PLoS One. 2017 May 18;12(5):e0177984. doi: 10.1371/journal.pone.0177984 (PMC5436893; doi:10.1371/journal.pone.0177984)
Supplement: S4 Table — (DOCX) [file pone.0177984.s004.docx]

**S4 Table: Calculation of vaccine contribution to mortality (example for MV as last vaccine)**

| Age group | Vaccination | Relative mortality | Proportion with most recent vaccine | |
| --- | --- | --- | --- | --- |
|  |  |  | 1992-93 | 2002-2003 |
| 1.5 – 8 months | DTP unvaccinated | 1(Ref) | 10% | 7% |
|  | DTP as most recent vaccine | 2.00 (1) | 90% | 93% |
| **Contribution of DTP to mortality in 2002-3 relative to 1992-3^b^** |  |  | **1 (Ref)** | **1.02** |
| 11 months | DTP as most recent vaccine | 1 (ref) | 33% | 25% |
|  | MV as most recent vaccine | 0.54 (Boys=0.87 , Girls =0.21)^a^ | 30% | 41% |
|  | DTP>=MV as most recent vaccine | 2.3*(0.54) | 31% | 28% |
| **Contribution of MV to mortality in 2002-3 relative to in 1992-3^c^** |  |  | **1 (Ref)** | **0.94** |

a: MV is associated with an estimated 46% reduction in mortality (2). Thus, we used 0.54 (1-0.46) as factor for calculating the relative reduction in mortality, 0.21 (1-0.79) for girls and 0.87 (1-0.13) for boys.

b: Calculation $\frac{\left( (\left( \text{Proportion with DTP for 1-8 months respectively, in 2002-3} \right)\text{*(2.00)) + DTP unvaccinated*1.00} \right)}{\text{ (((Proportion with DTP for 1-8 months respectively, in 1992-3)*(2.00) + DTP unvaccinated *1.00))}}$

c: Calculation: $\frac{\left( Proportion with MV\text{-}only at 11 months respectively, in 2002\text{-}3)\text{*}\left( \text{1-0.46} \right)\text{+ }\left( \text{Proportion }\text{with}\text{ MV+DTP at 11 months respectively, in 2002-3} \right)\text{*}\left( \text{2.3-1} \right)\text{+ }\left( \text{Proportion with DTP or unvaccinated at 11 months respectively, in 2002-3} \right)\text{*(1} \right)}{\text{(Proportion with MV-only at 11 months respectively, in 1992-3)*(1-0.46)+ (Proportion with MV+DTP at 11 months respectively, in 1992-3)*(2.3-1)+ (Proportion with DTP or unvaccinated at 11 months respectively, in 1992-3)*(1)}}$

1. Aaby P, Ravn H, Benn CS. The WHO review of the possible non-specific effects of diphtheria-tetanus-pertussis vaccine. 2016.

2. Higgins J, Soares-Weiser K, Reingold A. Systematic review of the non-specific effects of BCG, DTP and measles containing vaccines Geneva: World Health Organization; 2014. Available from: <http://www.who.int/immunization/sage/meetings/2014/april/3_NSE_Epidemiology_review_Report_to_SAGE_14_Mar_FINAL.pdf?ua=1>
